# The Synthetic Biology Open Language (SBOL) Version 3: Simplified Data Exchange for Bioengineering

**DOI:** 10.3389/fbioe.2020.01009

**Published:** 2020-09-11

**Authors:** James Alastair McLaughlin, Jacob Beal, Göksel Mısırlı, Raik Grünberg, Bryan A. Bartley, James Scott-Brown, Prashant Vaidyanathan, Pedro Fontanarrosa, Ernst Oberortner, Anil Wipat, Thomas E. Gorochowski, Chris J. Myers

**Affiliations:** ^1^School of Computing, Newcastle University, Newcastle-upon-Tyne, United Kingdom; ^2^Raytheon BBN Technologies, Cambridge, MA, United States; ^3^School of Mathematics and Computing, Keele University, Keele, United Kingdom; ^4^Computational Bioscience Research Center, King Abdullah University of Science and Technology, Thuwal, Saudi Arabia; ^5^Nuffield Department of Population Health, University of Oxford, Oxford, United Kingdom; ^6^Microsoft Research, Cambridge, United Kingdom; ^7^Department of Biomedical Engineering, University of Utah, Salt Lake City, UT, United States; ^8^Lawrence Berkeley National Laboratory, DOE Joint Genome Institute, Berkeley, CA, United States; ^9^School of Biological Sciences, University of Bristol, Bristol, United Kingdom; ^10^Department of Electrical, Computer, and Energy Engineering, University of Colorado, Boulder, CO, United States

**Keywords:** synthetic biology, data standards, data exchange, knowledge representation, SBOL

## Abstract

The Synthetic Biology Open Language (SBOL) is a community-developed data standard that allows knowledge about biological designs to be captured using a machine-tractable, ontology-backed representation that is built using Semantic Web technologies. While early versions of SBOL focused only on the description of DNA-based components and their sub-components, SBOL can now be used to represent knowledge across multiple scales and throughout the entire synthetic biology workflow, from the specification of a single molecule or DNA fragment through to multicellular systems containing multiple interacting genetic circuits. The third major iteration of the SBOL standard, SBOL3, is an effort to streamline and simplify the underlying data model with a focus on real-world applications, based on experience from the deployment of SBOL in a variety of scientific and industrial settings. Here, we introduce the SBOL3 specification both in comparison to previous versions of SBOL and through practical examples of its use.

## 1. Introduction

Synthetic biology builds upon advances in genetics, molecular biology, metabolic engineering, and other related disciplines by applying principles such as modularization, standardization, and a design-build-test-learn workflow to enable the engineering of biological systems, just as software engineering does to the design of computer programs (Endy, 2005). The design-build-test-learn workflow is heavily dependant on data exchange. A standardized knowledge representation, or data standard, for exchanging information is critical from the initial stage of knowledge gathering—where data about existing biological parts and systems must be integrated into a common model—through to the entire design-build-test-learn lifecycle. Data standards are also crucial for the effective dissemination of final products or the publication of novel designs to ensure precise and unambiguous details of a system are accessible for oversight, management, and potential future re-use.

The unique requirements of synthetic biology present a major barrier to the development of such standards. Biological designs often involve engineering activities across a wide range of scales, from single molecules to genes, pathways, strains, and complex multi-cellular systems. Consequently, synthetic biologists need to exchange a wide variety of information, including the intended behavior of the system and actual experimental measurements. Information being exchanged also often covers multiple aspects of a design, including nucleic acid sequences (e.g., the sequence that encodes an enzyme or transcription factor), molecular interactions that a designer intends to result from the introduction of a chosen sequence (e.g., chemical modification of metabolites or regulation of gene expression), as well as details regarding the construction of the final engineered strain (e.g., nucleic acid synthesis, assembly, and the transformation of a chosen cell type) and associated experiments and data. All of these diverse perspectives need to be effectively integrated to facilitate the effective engineering of biological systems.

While there already exist many computational representations of biological entities, these are almost all designed for the annotation of natural systems and therefore struggle to describe the specifics of engineered designs. For example, simple formats for representing sequences such as FASTA (Pearson, 1990) are focused purely at the scale of nucleic or amino acid sequences and cannot capture higher-level aspects of a design (e.g., a sequence composition from constituent sub-sequences/parts). More sophisticated formats such as GenBank (Benson et al., 2013) or GFF (Stein, 2013) provide a flat representation of sequence features that is well-suited to describing natural systems, but again are fundamentally focused on annotation at the nucleic or amino acid level and are therefore unable to effectively represent functional relationships between regions of a sequence (e.g., description of protein-protein interactions) and localization (e.g., intracellular transport, cell-to-cell communication), not to mention engineering concepts such as interfaces and specifications or information capturing the intent of the designer.

The *Synthetic Biology Open Language* (SBOL) has been developed to address these challenges. SBOL is a standard to support the specification and exchange of biological design information in synthetic biology (Galdzicki et al., 2014), following an open community process involving both “wet” bench scientists and “dry” scientific modelers and software developers across academia, industry, and other institutions (see Methods). One of the primary aims motivating the development of SBOL is the need to make the knowledge involved in the synthetic biology lifecycle computationally tractable and therefore amenable to process automation. The research question of how domain knowledge can be decomposed into a form accessible to computational methods is long-established in computer science. The Resource Description Framework (RDF) (W3C, 2014) is a data model formalized by the World Wide Web Consortium (W3C) to describe named properties and their values that is already widely used by the bioinformatics community, with some of the largest biological datasets such as UniProt and PubChem publishing official RDF versions (Redaschi and UniProt Consortium, 2009; Fu et al., 2015). SBOL is built upon RDF, and is also backed by a formally defined ontology (Misirli et al., 2019), allowing design data to be machine-navigable as a knowledge graph.

Since its initial publication in 2011, SBOL has become the recommended format for engineered nucleic acid constructs in ACS Synthetic Biology (Hillson et al., 2016), and is supported by many biological design tools. For instance, Eugene (Bilitchenko et al., 2011; Oberortner et al., 2014; Oberortner and Densmore, 2015), GEC (Pedersen and Phillips, 2009; Dalchau et al., 2019), Cello (Vaidyanathan et al., 2015; Nielsen et al., 2016), GenoCAD (Czar et al., 2009), ShortBOL (Crowther et al., 2020), and GeneTech (Baig and Madsen, 2017) provide computational frameworks for combinatorial design space exploration, where users can specify structural, functional, and performance constraints. The outputs generated by these tools in SBOL can then be directly used by DNA assembly planning software tools such as BOOST (Oberortner et al., 2017), Raven (Appleton et al., 2014), j5 (Hillson et al., 2012), and DeviceEditor (Chen et al., 2012) to automate the process of physically building DNA constructs. Tools such as iBioSim (Myers et al., 2009; Watanabe et al., 2018), MoSeC (Misirli et al., 2011), and SBOLDesigner (Zhang et al., 2017) support the same SBOL data format and support the modeling, analysis, and simulation of biosystems. There are also a number of data repositories, registries, and databases that support and store data in the SBOL format, such as SynBioHub (McLaughlin et al., 2018), SBOLme (Kuwahara et al., 2017), JBEI-ICE (Ham et al., 2012), and the Virtual Parts Repository (VPR) (Cooling et al., 2010; Hallinan et al., 2014; Misirli et al., 2014). The SBOL community has also developed a graphical language for the visualization of biological designs (Quinn et al., 2015; Beal et al., 2019), which has been used in combination with the data standard in tools such as Pigeon (Bhatia and Densmore, 2013), DNAPlotlib (Der et al., 2017; Bartoli et al., 2018), VisBOL (McLaughlin et al., 2016), Constellation, and SBOLCanvas. These tools help to visualize constructs in the computational synthetic biology space such as genetic circuits, biochemical components, and possible design spaces based on structural or functional constraints. There are many other examples that highlight the utility of the SBOL data exchange format to connect and integrate data to create a seamless computational workflow. For instance, Cello (Nielsen et al., 2016) adopted the concept of a User Constraint File (UCF) used in digital logic design to specify the library of genetic gates and the associated properties and meta-data required to synthesize combinational Boolean logic circuits. In addition to this UCF file, the same library is also available in SBOL format, which allows the data from Cello to be used in other tools and workflows as highlighted in a recent effort to use the Cello library and Virtual Parts Repository API to build computational models encoded in the *Systems Biology Markup Language* (SBML) (Hucka et al., 2003) that could be simulated using iBioSim (Misirli et al., 2018).

**Figure 1:**
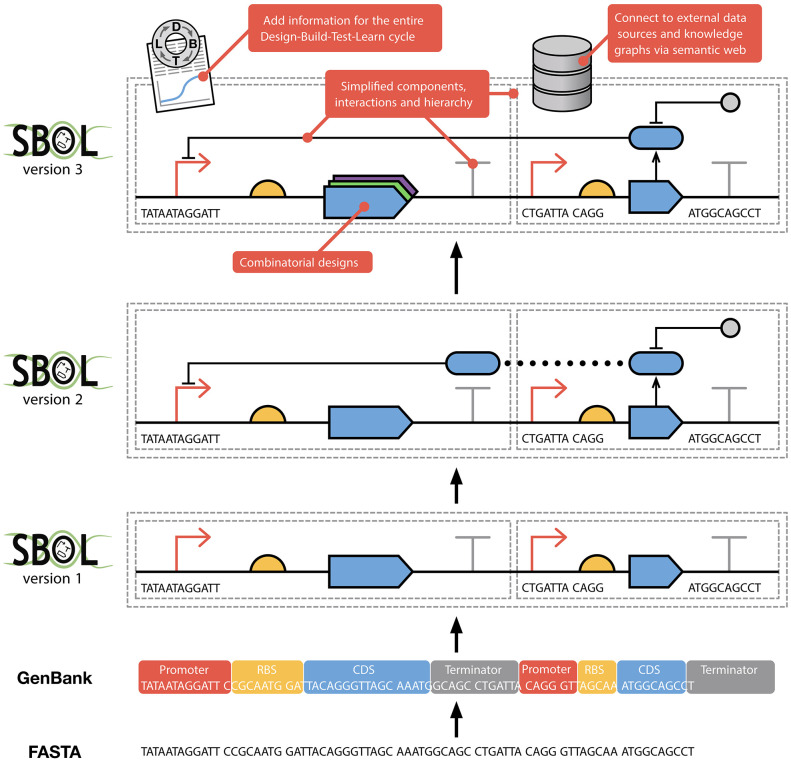
The evolution of SBOL from earlier FASTA and GenBank formats. FASTA was developed to capture pure sequence information. GenBank extends upon this allowing sequences to have annotations, thereby capturing some structural and functional information. SBOL1 adds the ability to use hierarchical composition when describing a design as well as only partially specifying sequences. The complementary SBOL Visual standard (Beal et al., 2019; Madsen et al., 2019a) enables the visual representation of biological design information in an unambiguous way (SBOL version 1, 2, and 3 designs are all shown using SBOL Visual). SBOL2 added the ability to specify modules and functional interactions between parts. Finally, SBOL3 simplifies the SBOL2 data model and greatly improves interoperability with other computational tools through the use of a standardized knowledge graph representation.

The first version of SBOL (Galdzicki et al., 2011) defined a simple data model for the description of engineered DNA components and their sequences. Since then, SBOL has evolved to support the capture of information at many different levels of representation across entire synthetic biology workflows (Figure 1). In particular, the previous major revision, SBOL2 (Bartley et al., 2015; Roehner et al., 2016), generalized the data model to allow for designs to include not only DNA components, but also other molecular species such as RNAs, proteins, larger components of a system such as whole cells, and links to models encoded using complementary standards such as SBML (Hucka et al., 2003). The standard was also incrementally expanded with several minor revisions (Beal et al., 2016; Cox et al., 2018; Madsen et al., 2019b) to capture information about combinatorial design libraries, external file attachments, sequence construction, experimental tests, and measurements. Furthermore, by leveraging the Provenance Ontology (PROV-O) (Lebo et al., 2013), SBOL2 can capture provenance information to link and trace information and processes throughout the entire design-build-test-learn cycle.

The incremental expansion in the scope of SBOL2 over the past few years has resulted in a significant increase in the complexity of the SBOL data model and has revealed aspects of the representation that limited future developments. While SBOL Enhancement Proposals (SEPs) to address this complexity had been accepted by the community, they were considered too major for a 2.x release, and therefore the need for a new major iteration of SBOL became apparent.

Here, we present SBOL version 3 (SBOL3), a substantially simplified standard that addresses these limitations, building upon the experience of the SBOL community applying SBOL across scientific and industrial settings. This new version (Baig et al., 2020) provides for a more direct and elegant expression of the diverse types of biological design information in use today, while at the same time reducing the complexity of the data model, which helps simplify the development of supporting libraries and data exchange with compatible tools. SBOL3 is an attempt to learn from the application of the previous SBOL standards, take stock of new developments and directions in the field, and establish a strong foundation for improved data exchange and computational-accessibility across synthetic biology.

## 2. Results

**Figure 2:**
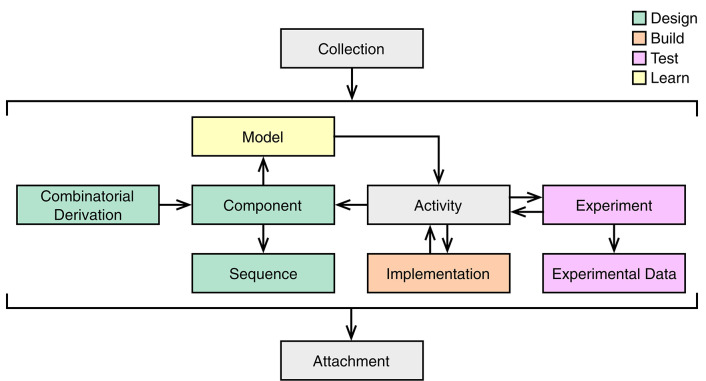
Main top-level classes of SBOL3 and their relationships. The color of each class corresponds to its role in the design (green), build (orange), test (pink), and learn (yellow) stages of the synthetic biology workflow. Additional utility classes are represented by gray boxes.

SBOL3 contains ten main top-level classes to support the various aspects of the design-build-test-learn workflow (Figure 2). In particular, designs can be expressed using the Component, Sequence and CombinatorialDerivation classes. The Component class is intended to be widely applicable across all scales of biodesign, and can be used to describe not only genetic designs, but also the design of other biological entities such as proteins, functional RNAs, strains, multicellular systems, media, and experimental samples. For those Components that have a defined primary structure, such as nucleic acids and proteins, an instance of the Sequence class can be assigned. A CombinatorialDerivation allows one to specify a design pattern where individual SubComponents can be selected from a set of variants.

Beyond design, the Implementation class corresponds to the build stage of the synthetic biology lifecycle and is used to represent physical entities such as a sample of plasmid, a stab of transformed bacteria, or an aliquot of liquid culture. The Experiment and ExperimentalData classes support the test stage, allowing for the linking of data generated during an experiment. The Model class associates learned information with a design. All of this information can be linked together using the Activity class from PROV-O (Lebo et al., 2013). For example, a *design* Activity may describe how a Component is designed from a Model description. A *build* Activity describes how an Implementation is constructed to the specification of a Component description. A *test* Activity describes how an Experiment is conducted using an Implementation artifact. Finally, a *learn* Activity may describe how a Model is updated using information from an Experiment. The Collection class has members which can be of any of these types or even Collections themselves. Finally, all of these objects can refer to objects of the Attachment class, which is used for links to external data (images, spreadsheets, textual documents, experimental instrument outputs, etc.).

### 2.1. SBOL3 Components

**Figure 3:**
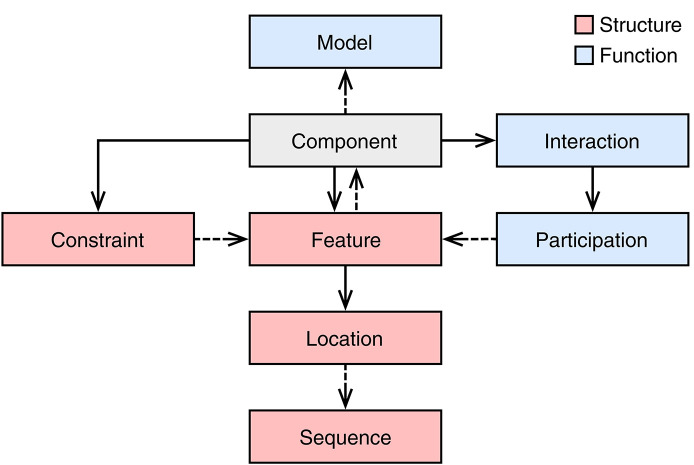
The SBOL3 Component object and related objects. Solid arrows indicates ownership and dashed arrows represent a reference to an object of another class. Red and blue boxes represent structural and functional objects, respectively. To represent structural aspects, a Component can include Features, which may refer to Locations within a Sequence. A Component can also include Constraints between these features. To represent functional aspects, a Component can include Interactions that can refer to relationships between participating Features. A Component can also have its behavior described using a Model.

The main design entity in SBOL3 is the Component class. Figure 3 provides an overview of the classes used by or linked to by the Component class. The “structural” classes have existed in various forms since the original SBOL1 specification. SBOL2 introduced the “functional” classes of Interaction, Participation, and Model. When SBOL2 introduced these classes, they were intentionally kept separated from structural information in a parallel “module” class hierarchy, with the aim of allowing a simpler core “component” hierarchy to focus on the construction of nucleic acid sequences and to be largely shared with SBOL1. As SBOL has been applied to an expanding range of designs, engineering scales, and workflows, however, it has become clear that this dichotomy often tended to create additional complexity by separating elements of a design that would more naturally exist in the same scope. A summary of the changes to the “component” hierarchy is provided in Table 1.

**Table 1:** Table of usage scenarios and their corresponding classes in SBOL version 1, 2, and 3.

	**SBOL1**	**SBOL2**	**SBOL3**
DNA part	DnaComponent	ComponentDefinition	Component
Non-DNA part	N/A	ComponentDefinition	Component
Part uses	SequenceAnnotation	Component	SubComponent
Functional groups	N/A	ModuleDefinition	Component
Func. group uses	N/A	Module	SubComponent
Sequence features	SequenceAnnotation	SequenceAnnotation	SequenceFeature
References	N/A	MapsTo	ComponentReference
External definitions	N/A	N/A	ExternallyDefined
Placeholders	N/A	N/A	LocalSubComponent

**Figure 4:**
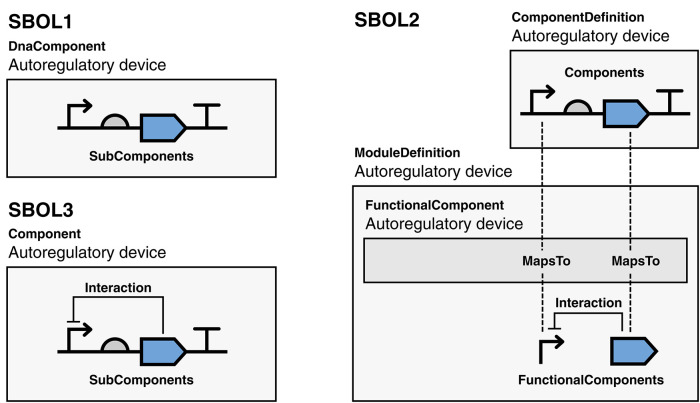
A simple auto-regulatory device represented using SBOL versions 1, 2, and 3. In the SBOL1 example, the structure of the unit is captured, but the regulatory function is not. In the SBOL2 example, the structure is captured using a ComponentDefinition, the function is captured in a separate ModuleDefinition, and the two objects are connected using MapsTo relations. In the SBOL3 example, both the structure and function are captured by a single Component, under which the SubComponent and Interaction objects can co-exist. Diagrams are drawn using SBOL Visual notation (Beal et al., 2019).

For example, consider a simple auto-regulatory device: a transcriptional unit comprising a promoter, ribosome binding site (RBS), coding sequence (CDS), and terminator, where a transcription factor encoded by the CDS represses the activity of the promoter (Figure 4). In SBOL1 (or an annotation format such as GenBank or GFF), only the genetic structure of the transcriptional unit can be represented, omitting the regulatory relationship. An SBOL2 representation begins similarly, with a ComponentDefinition to represent the transcriptional unit as a whole, with its parts each a Component instantiations of the ComponentDefinition for the respective constituent promoter, RBS, CDS, and terminator parts, with these functions identified using terms from the *Sequence Ontology* (SO) (Eilbeck et al., 2005). The auto-regulatory interaction must then be expressed separately in a ModuleDefinition which, like the ComponentDefinition, describes the transcriptional unit, but this time, from a functional perspective. To do this, the transcriptional unit must be instantiated in the ModuleDefinition using a FunctionalComponent, but its parts are still contained within the ComponentDefinition and are not exposed at the level of the ModuleDefinition. To document the interaction, therefore, it is also necessary to create promoter and CDS FunctionalComponent objects at the level of the ModuleDefinition and a MapsTo relation for each that identifies the promoter and CDS in the ModuleDefinition as being the same promoter and CDS in the ComponentDefinition. Finally, an Interaction can be created in the ModuleDefinition to indicate that the CDS has a regulatory effect on the promoter. While this representation does capture all of the information desired, synthetic biologists do not typically separate their thinking in this manner: the promoter and CDS are being composed as they are in the sequence structure precisely because of their expected interaction. As a result, rather than deriving advantage from the separation, SBOL tools instead tend to try to hide the distinction from the user, further increasing both complexity and opportunity for error.

In SBOL3, structural and functional aspects are both captured using a single Component class (Figure 3). Namely, to represent structural aspects, a Component can include Features, some of which may be at some Location within a Sequence, and which may have Constraints expressing other relations in identity or space. To represent functional relationships a Component can include Interactions that can refer to relationships between participating Features. Finally, a Component can refer to an externally defined model using the Model class. The SBOL3 representation in Figure 4 shows how much simpler this unified approach can be, with the functional information added through a single Interaction rather than an entire parallel construct and set of identity mappings.

**Figure 5:**
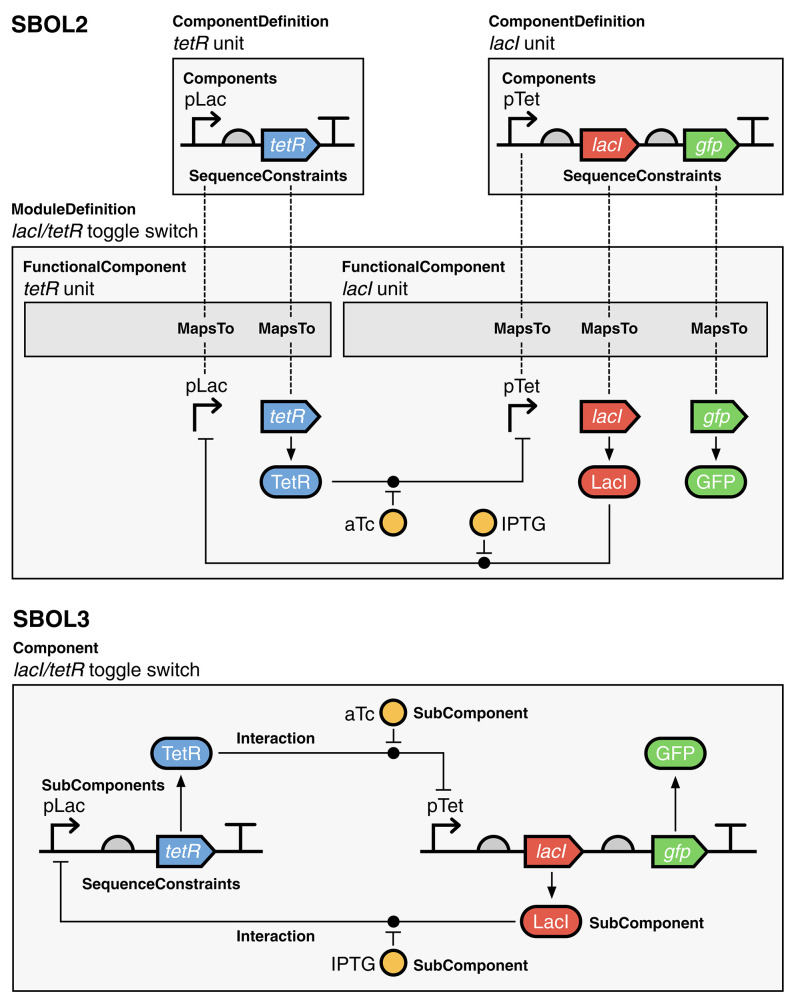
Gardner et al. (2000) toggle switch represented using SBOL versions 2 and 3. In the SBOL2 representation, the structure of the *lacI* and *tetR* units are defined in ComponentDefinitions and instantiated in a ModuleDefinition as FunctionalComponents. Their function-linked sub-structures are then mapped into the ModuleDefinition using MapsTo to assign corresponding FunctionalComponents for the promoter and CDS components, which can then be used as participants in Interactions. In the SBOL3 representation, the creation of a ModuleDefinition and MapsTo relations as in the SBOL2 example is no longer necessary as sequence information and interactions can co-exist in the same parent Component object. Diagrams are drawn using SBOL Visual notation (Beal et al., 2019). A serialized SBOL3 representation of this construct is available on GitHub at https://github.com/SynBioDex/SBOLTestSuite/tree/master/SBOL3/toggle_switch.

A more complex example illustrating the advantage of this approach is shown in Figure 5 for the classic genetic toggle switch (Gardner et al., 2000). As with the auto-regulatory device, the SBOL2 representation has compact structural representations of each transcriptional unit, but the functional representation “explodes” these back into a collection of copies and identity mappings for all of the elements that participate in interactions. In SBOL3, on the other hand, the combination of structural and functional information into a single Component means that every element of the system appears precisely once and no identity mappings are necessary.

**Figure 6:**
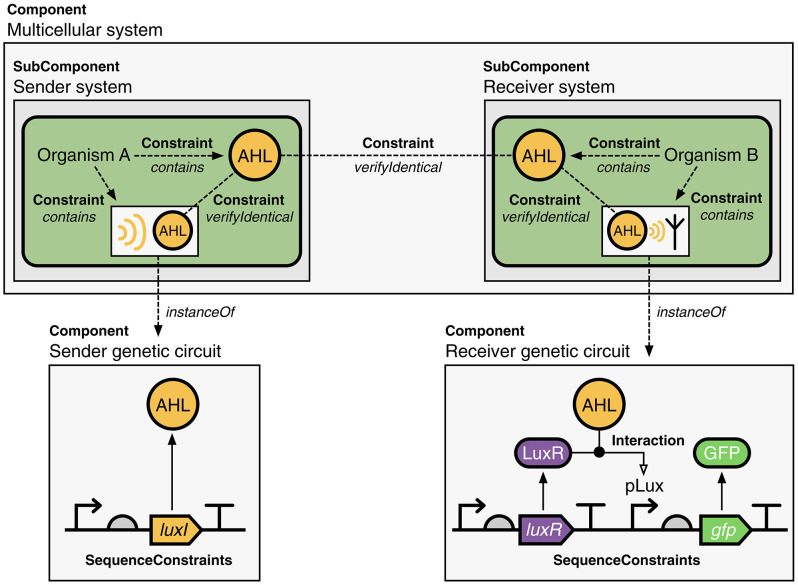
A multicellular communication system represented using SBOL3. Two different organisms implement a sender and receiver system, which uses a small molecule (AHL) as a signal. The sender and receiver systems are represented by Components and use constraints to show that each of these cell types contains AHL (not shown are details of the genetic system and its interactions with the molecule). These sender and receiver systems are SubComponents of the overall multicellular system, which is also represented by a Component. The fact that AHL is shared between the two systems is captured using an identity constraint. A serialized SBOL3 representation of this construct is available on GitHub at https://github.com/SynBioDex/SBOLTestSuite/tree/master/SBOL3/multicellular.

The generalization of Component in SBOL3 enables a single, unified hierarchy to capture designs comprising components across multiple scales of a design, from individual molecules to entire cells. For example, the system depicted in Figure 6 illustrates how the SBOL3 Component class can be used to represent a multicellular system where a signaling molecule (AHL) is used for communication between “sender” and “receiver” cells. Moving to these larger scales is also enabled by expanding Component type information beyond the Sequence Ontology to additionally use appropriate classes of terms from the Systems Biology Ontology (SBO) (Courtot et al., 2011) and Gene Ontology (GO) (Harris et al., 2004). In this multicellular system, for example, each cell is assigned the role SBO:0000290 (physical compartment) and type GO:0005623 (cell), while the subsystems for the sender and receiver are each assigned the role SBO:0000289 (functional compartment). Constraints are then used to express the spatial structure of the systems, with the sender cells acting to produce AHL molecules initially contained within those cells, the receiver cells responding to the AHL molecules contained within those cells, and the fact that AHL is being shared between the two types of cells is represented by an identity relation between the two instances of the molecule.

Finally, to better support the expanded range of design elements that can be represented, SBOL3 also changes the ontology used for specifying the type of a Component. Previous versions of SBOL used the BioPAX (Demir et al., 2010) definitions for molecular species, such as DNA and protein ComponentDefinition instances. However, this set of species is restricted, making it difficult to describe designs across different molecular scales. The Systems Biology Ontology (SBO) (Courtot et al., 2011) provides a much richer and more extensible set of terms, already used by SBOL2 in the Interaction and Participation classes and by SBOL Visual. SBOL3 standardizes the definition of molecular species on SBO in order to have a more expressive and consistent specification of component types. For example, a DNA Component can be labeled using the SBO term SBO:0000251 (Deoxyribonucleic acid), while a complex can be labeled using SBO:0000254 (Non-covalent complex). A Component used to represent primarily functional rather than structural relationships, on the other hand, such as a metabolic synthesis pathway spanning multiple integration sites, uses the SBO:0000241 (Functional entity) term.

### 2.2. Features

In SBOL3, the Feature class is used to specify elements of interest within a Component. SBOL3 introduces several other classes of Feature to enable simpler representation of synthetic biology designs.

#### 2.2.1. SubComponents and SequenceFeatures

The original SBOL1 and SBOL2 structural representations focused on the hierarchical composition of parts, such as the inclusion of the *pBAD* promoter in the design of an arabinose sensor. This was accomplished in SBOL2 using a Component (now a subclass of Feature called SubComponent in SBOL3) to refer to a definition of the included part, while its location or locations on the sequence (if known) were expressed using a SequenceAnnotation.

However, there are many simpler features (such as a restriction site or -35 region) which are useful to annotate but do not have any meaningful separate hierarchical existence within a design. As SBOL2 evolved, such annotations were simplified by allowing a SequenceAnnotation to provide feature information about a sequence directly, without the need to link the annotation to a Component, but the two classes could not be separated fully without breaking backward compatibility.

In SBOL3, sub-components and feature annotation are now fully refactored into two separate subclasses of Feature. The SubComponent subclass describes a hierarchical part-subpart relationship, with the option to directly specify its location on a sequence if known and relevant, while the SequenceFeature subclass describes a feature that must be associated with a location, but does not indicate a part-subpart relationship.

#### 2.2.2. Local and External Design Elements

SBOL3 also simplifies the handling of two other common cases where defining a full Component is not useful. First, similar to SequenceAnnotation, a LocalSubComponent is used to represent components whose only purpose is to be local placeholders or composites that only really make sense within the context of their parent Component, being defined in terms of their relationships with other Features. For example, a LocalSubComponent may be used to specify a variable in a template for a combinatorial library, with the local subcomponent indicating information such as “put a promoter in this location” and “put a barcode in that location.” In another example, a LocalSubComponent can be used to specify a plasmid assembled from several SubComponents, which then goes on to be transformed into a cell strain.

Another important case is when an established collection of knowledge is better kept outside of SBOL entirely. For example, knowledge about small molecules or proteins is already thoroughly encoded in a standard format in databases such as ChEBI (Degtyarenko et al., 2007) or UniProt (UniProt Consortium, 2007). In SBOL3, an ExternallyDefined feature allows such elements to be included in a design by pointing to the canonical non-SBOL definition, while still giving sufficient information to reason about its use within a design via type and role properties from ontologies such as SBO and GO. In SBOL2, by contrast, such elements were required to be mirrored in “empty” ComponentDefinition objects that still essentially just served as a link to the definition while tending to obfuscate the sharing of common design elements.

#### 2.2.3. Simplified References

Finally, SBOL3 also introduces a ComponentReference class that allows a Feature within a SubComponent to be used directly in an Interaction or Constraint relationship. For example, a ComponentReference can be used in an Interaction indicating that the TetR protein represses the pTet promoter on a plasmid that is included in a design as a SubComponent.

This greatly simplifies such representations relative to SBOL2. In SBOL2, such a reference was constructed by importing a copy of the element as an immediate child of the object where the relationship was expressed and then linking this copy to the original with a MapsTo identity relation. The ComponentReference approach also enables multi-layer references, which were not possible in SBOL2 without also modifying the description of the intermediate layer designs.

### 2.3. Generalized Constraints

While the Interaction class can be used to express functional relationships between biological components, it is also often useful to be able to express information about the non-functional design relationship between components. Such relationships include identity (e.g., replacing a placeholder in a template with a complete definition), relative positions in a sequence (e.g., “pLac precedes *tetR*”), and general spatial relations (e.g., containment of a plasmid in the chassis strain it transforms). The incremental growth of SBOL2 resulted in this information being expressed in a limited manner across a mixture of different classes: identity relationships were expressed using a mix of MapsTo and SequenceConstraint objects, while spatial relationships were expressed with a mix of SequenceConstraint and Interaction objects. SBOL3 combines and generalizes these into a unified Constraint class, in which two components (a subject and an object) are linked using a restriction to express their relationship.

In SBOL3, identity relationships between components are expressed with the verifyIdentical, differentFrom, and replaces relationships. The SBOL2 relationships for expressing relative positions in a sequence—precedes, sameOrientationAs, and oppositeOrientationAs—are expanded with additional restrictions that cover the full range of sequential relationships (Allen, 1983): strictlyPrecedes, meets, overlaps, contains, strictlyContains, equals, finishes, and starts.

Likewise, the set of constraints is further expanded to deal with the spatial relationships of physical objects in general, rather than just the special case of directional linear sequence. In particular, these relations are based on the set of all topological relationships between two spatial regions without holes (Egenhofer and Herring, 1991), including common unions and omitting symmetric relations that can be expressed by swapping subject and object. These new topological restrictions include:

- isDisjointFrom – subject and object do not overlap in space. Example: a plasmid is disjoint from a chromosome.
- strictlyContains – subject entirely contains object: they do not share a boundary. Example: a cell contains a plasmid.
- contains – subject contains object and they might or might not share a boundary. Example: a cell contains a protein that may or may not bind to its membrane.
- meets – subject and object are connected at a shared boundary. Example: two strains of adherent cells meet at their membranes.
- covers – subject contains object but also shares a boundary. Example: a bacterial cell encloses its transmembrane proteins.
- overlaps – subject and object overlap in space, but portions of each are outside of the other. Example: a transmembrane protein overlaps the cell membrane.

Taken all together, these three sets of relationships provide a much simpler and more expressive system for expressing design constraints in SBOL3 than existed in SBOL2.

### 2.4. Interfaces

In SBOL2, information about the recommended interface for a component/module was dispersed into the “access” field of ComponentInstance and the “direction” field of FunctionalComponent. This makes the interfaces implicit rather than explicit, scatters the information, and forced premature definition of information about interfaces. As SBOL is now being used to build designs that comprise more complex devices on a larger scale, a clear specification of how components work together is highly important.

In SBOL3, this information is instead collected into an explicit Interface object with input, output, and non-directional properties. Each of these properties refers to a set of Feature objects in the same Component that owns the Interface. Specifying any Interface is optional, however, so this information only need be added to systems where it makes sense and at an appropriate stage of engineering. For example, a NOR gate from (Gander et al., 2017) could be described as an SBOL3 Component with four SubComponents: two gRNA inputs, the DNA component that they regulate (comprising two binding sites, a promoter, and a gRNA coding sequence), and the gRNA output. It would then be assigned an Interface with two input relations (to the input gRNA SubComponents) and one output relation (to the output gRNA SubComponent).

### 2.5. Relationship With RDF and the Semantic Web

All versions of SBOL have used RDF as a serialization format. However, the relationship between SBOL and its underlying Semantic Web representation has previously been unclear. SBOL3 addresses these issues by following Semantic Web related best practices where possible, enabling better integration with existing Semantic Web tools.

#### 2.5.1. Consistent Property Names

SBOL uses many terms from existing ontologies, such as Dublin Core and PROV-O. The SBOL1 and SBOL2 specifications were written in a manner such that those terms were given a new “SBOL alias” that was sometimes, but not always, distinct from the name assigned to them by the ontology. For example, instead of defining the concept of a “title” or “description,” the SBOL2 specification used the dcterms:title and dcterms:description properties from the Dublin Core ontology. However, the dcterms:title property is first introduced as the “SBOL alias” of name, and then later “mapped” to an ontology term in the serialization section of the specification.

This makes serialized SBOL confusing to read, because the ontologically-defined names used in the serialization do not always match the specification-defined names used by SBOL libraries. For example, SBOL2 renames the prov:wasDerivedFrom property to wasDerivedFroms for consistency with other aliases used in the specification. This also meant that integrating terms from other ontologies into SBOL2 required a two-step process of writing their description as SBOL “aliases” and then writing their “serialization.”

In SBOL3, the use of external ontologies has been made explicit and consistent throughout the specification. For example, dcterms:title has been replaced with an sbol:name property, and all of the diagrams in the specification have been updated to display the singular, prefixed form of property names (e.g., prov:wasDerivedFrom) rather than an “SBOL-adjusted” version (wasDerivedFroms).

#### 2.5.2. Differentiating SBOL Entities (Concepts) and Properties

The SBOL2 data model has several labels that are both used to refer to entities and property names. In SBOL2, they were differentiated by using the uppercase letter when referring to entities and using the lowercase letter when referring to property names. However, not all RDF tools are case-sensitive. Moreover, referring to the data model makes it more difficult to explain in papers. In SBOL3, this ambiguity is removed and the labels are made as unique as possible. Additionally, prefixing and suffixing is applied to property names, e.g., “has.” or “is.Of,” as is the Semantic Web convention. For example, the SBOL2 interaction property is now hasInteraction in SBOL3. Additionally, all entities that are represented as RDF resources now begin with an uppercase letter, again following RDF convention. For example, the public specifier in SBOL2 is now Public in SBOL3.

#### 2.5.3. Serialization

Before SBOL3, the standard specified a bespoke file format used for data exchange. This file format required the development of libraries specifically for serializing and parsing SBOL data. In contrast, SBOL3 no longer specifies a particular file format for data exchange. Rather, it specifies how SBOL data structures map to an RDF graph representation. This graph may then be easily serialized to and parsed from a number of file formats, such as XML, Turtle, N-Triples, and JSON, using standard software packages. In addition to simplifying the underlying software implementation, different serialization formats may provide advantages for certain users. For example, Turtle increases human-readability of SBOL documents, and even allows them to be edited manually, while JSON is particularly convenient when developing web applications using JavaScript, and N-Triples is better for minimal difference detection version control systems.

### 2.6. Namespaces and Identifiers

Finally, one of the important considerations for enabling design data interoperability is the need for consistent and compatible identifiers. As SBOL is built upon RDF, it inherits the World Wide Web concept of a *Uniform Resource Identifier* (URI), a superset of the *Uniform Resource Locator* (URL) standard. Consequently, most SBOL resources, whether in a local SBOL file or in an online repository, have an identifier that resembles a Web address.

In SBOL1, the format of these URIs was left unspecified, meaning there is little consistency in the URIs created by different SBOL1-enabled tools. SBOL2 introduced the concept of “compliant URIs,” which comply with a set of optional best practice rules. Broadly, compliant URIs take the form of <URI prefix>/<displayId>/<version>, where the URI prefix of child objects must be prefixed with the persistent identity of their parent.

While SBOL2 compliant URIs are an improvement over the lack of specification in SBOL1, they also suffer from several practical issues. First, the positioning of the version at the end of the URI is contrary to the established RDF convention of positioning the identifier at the end, meaning existing RDF tooling often displays the version of SBOL2 resources in place of the identifier. Second, URI-suffix versioning is too granular (at object level, when changes are often made across many objects in a design) but also too contagious (changing an object version requires making duplicate copies of everything that points to it as well). Finally, these rules remain optional, meaning there is no guarantee that SBOL2 data has compliant URIs, and it is unclear when implementing tooling how to handle the case of mixed compliant and non-compliant URIs.

SBOL3 addresses these issues by replacing the best practice of compliant URIs with a required SBOL3 URI structure of the form <URI prefix>/<displayId>, leaving the handling of versioning and placement (if any) of the version up to the tooling. For example, the version could become part of the prefix (e.g., http://example.com/toggleswitch/1/lacI, part of the displayId (e.g., http://example.com/toggleswitch/lacI_1, or even omitted entirely (e.g., handled instead via git versioning).

Another challenge in SBOL2 was determining which portion of a URI to rewrite when moving it from one namespace to another. This often occurs when an SBOL document is migrated from hosting on one server to a new location on a different server, due to the dual role of a URI as both identifier and Web locator. SBOL3 addresses this by introducing a Namespace class that can be used to explicitly encode which portion of a set of URIs should change and which should be retained.

## 3. Discussion

SBOL supports the representation of abstraction hierarchies across multiple scales of bioengineering, from individual molecules to multi-cellular compositions and complete synthetic genomes (Bartley et al., 2020). The SBOL data model supports a wide variety of important use cases for synthetic biology and bioengineering, including visualization (McLaughlin et al., 2016), sequence design automation (Zhang et al., 2017), sharing of genetic design information (McLaughlin et al., 2018), metabolic engineering (Kuwahara et al., 2017), and generation of dynamical models from sequence representations (Misirli et al., 2018). Additionally, SBOL can be used to capture information about the workflows used to engineer biological systems, supporting reproducibility and automation of these processes.

As described in this paper, the SBOL community has drawn upon several years of experience with the real-world use of SBOL in scientific and industrial settings to produce a specification for SBOL3 that is simultaneously simpler and more expressive. Improvements to the standard in SBOL3 generally fall into one of two categories: simplification of the data model, or closer conformance with Semantic Web best practices. Major simplifications in the data model include the unification of structural and functional compositions into a single component hierarchy; simplification of the description of sub-components and sequence features; and simplifying connections between inputs and outputs across modular interfaces (e.g., transcriptional logic gates).

The other category of improvements in SBOL3 adjust the standard to take better advantage of Semantic Web technologies. By embracing existing developments, this shift will enable more rapid development of SBOL tools and libraries and simplify their maintenance. It will also enable users of SBOL to more easily integrate biological knowledge in the context of their tools through the use of ontologies, which are already widely used in the life sciences to explicitly define biological entities and their relationships. In addition to building upon existing ontologies wherever possible such as the Sequence Ontology (Eilbeck et al., 2005) and the Systems Biology Ontology (Courtot et al., 2011), SBOL itself is now represented as a machine-readable ontology, SBOL-OWL (Misirli et al., 2019). Similar to how ontologies are built upon the RDF layer to provide the meaning of RDF graphs, SBOL-OWL defines data model entities that are used to build SBOL graphs. Formal representation of the data model as an ontology opens up the possibility of using different Semantic Web tools, such as using existing reasoners to infer information, or validating SBOL data against a schema. Logical axioms are then used to constrain how different SBOL entities can be used together. SBOL-OWL is also embedded into the SBOL Visual Ontology (Misirli et al., 2020), which has been developed as a machine-accessible catalog of glyphs. This integration further facilitates searching for standard SBOL glyphs using ontological terms, and a web service layer enables accessing these glyphs via the Internet.

Overall, these improvement produce a new version of SBOL that provides for a more direct and elegant expression of a broad range of bioengineering information, while at the same time reducing the number of complex classes and rules to a functional minimum, thus providing a significantly improved means of data exchange. These improvements will thus facilitate easier adoption by new users and more rapid development of software tools and datasets that make use of the standard.

### 3.1. Future Work

The dramatic expansion in scope from the simple DNA components of SBOL1 to the complex systems across multiple scales captured by SBOL3 was driven by the needs of the synthetic biology community, as the field of synthetic biology matured and its applications became both more widespread and more complex. The SEP process by which SBOL3 was developed ensures the standard can continually adapt to the changing requirements of an evolving discipline, while ensuring that proposed changes are ratified by the community. For example, proposals for SBOL 3.0.1 have already been made to improve internationalization by adopting a file encoding and replacing Uniform Resource Identifiers (URIs) with Internationalized Resource Identifiers (IRIs).

While the nature of future requirements can only be speculated, there are many aspects of the synthetic biology lifecycle which remain largely unspecified by SBOL. For example, while SBOL recommends the use of the prov:Plan class, it does not yet recommend any domain-specific properties for its annotation. Equally, while the concept of an experiment can be captured in SBOL, it does not yet standardize metadata about the experiment or experimental data. Future revisions of the SBOL standard will therefore undoubtedly concern not only its expressiveness in describing design elements, but also its ability to capture and formalize the synthetic biology lifecycle as a whole.

## 4. Methods

Since its inception, the SBOL Standard has been developed as a community effort by the SBOL Development Group, which is open to any interested person. However, the development process was largely informal until the SBOL Enhancement Proposal (SEP) mechanism was introduced in 2015 (Grünberg and Bartley, 2015), shortly after the finalization of the SBOL2 specification. Development of SBOL3 has been driven by this formal process of documenting user experiences, developing proposals, and constructively debating the merits of these proposals.

Under this process, any SBOL user can propose a change by drafting a document with a specific format (an SEP), which is then discussed by the community on the mailing list and in GitHub issues associated with the SEP. Once the current elected editors of the standard judge that an SEP has been discussed sufficiently and an approximate consensus achieved, a voting form is posted, and any member of the SBOL Developers Group can vote for or against it. The SEP is immediately accepted if at least a two-thirds majority of votes cast are in favor. Otherwise, there is a further period of discussion, during which the SEP can be modified or withdrawn by its original author(s), followed by a second vote in which only a simple majority is required for acceptance.

Since the publication of SBOL2 in 2015, 46 SEPs have been opened, as community experience in deployment of SBOL revealed some of the practical challenges and opportunities for enhancement. Of these SEPs, twelve were implemented as incremental updates to SBOL2, resulting in significant milestones in SBOL version 2.1.0 (Beal et al., 2016), which introduced feature annotation and the encoding of provenance information to trace the history of designs; SBOL version 2.2.0 (Cox et al., 2018), which introduced support for combinatorial designs; and SBOL version 2.3.0 (Madsen et al., 2019b), which introduced extensions to support measurements, parameters, and the organization and attachment of experimental data.

Other SEPs were deemed too major to be integrated into a 2.x release of SBOL, since they would create backwards compatibility problems. Therefore, they were scheduled for SBOL version 3. After a series of community votes, a working group met to assemble the SBOL3 specification at the HARMONY 2020 Workshop at EMBL-EBI in Cambridge, UK. The resolution of conflicts between these SEPs resulted in a final SEP summarizing all changes in the SBOL3 data model. After voted acceptance of this SEP by the community, the SBOL3 specification was finalized.

Though there are not yet any complete software implementations of SBOL3, the SBOL community has established an SBOL3 implementation working group comprising many of the developers of libraries for previous SBOL versions and other interested parties. The first software libraries are expected to be released within the coming months for Java, Python, and JavaScript. Preliminary support for SBOL3 has been implemented in ShortBOL (Crowther et al., 2020), a tool for composing SBOL using a shorthand syntax.

## Data Availability Statement

Publicly available datasets were analyzed in this study. The specifications described in this article are freely available from https://sbolstandard.org/data-model-specification/.

## Author Contributions

JM, JB, GM, RG, and CM authored the SEP proposals from which the SBOL3 standard was developed. JM, JB, GM, RG, BB, JS-B, TG, PV, PF, EO, AW, and CM developed the SBOL3 specification and authored this manuscript. All authors contributed to the article and approved the submitted version.

## Conflict of Interest

The authors declare that the research was conducted in the absence of any commercial or financial relationships that could be construed as a potential conflict of interest. The handling editor declared a past co-authorship with one of the authors TG.
